# First-sight recognition of touched objects shows that chicks can solve Molyneux's problem

**DOI:** 10.1098/rsbl.2024.0025

**Published:** 2024-04-03

**Authors:** Elisabetta Versace, Laura Freeland, Michael G. Emmerson

**Affiliations:** Department of Biological and Experimental Psychology, School of Biological and Behavioural Sciences, Queen Mary University of London, 327 Mile End Road, London E1 4NS, UK

**Keywords:** chicks, cross-modal cognition, tactile, imprinting, visual, Molyneux's problem

## Abstract

If a congenitally blind person learns to distinguish between a cube and a sphere by touch, would they immediately recognize these objects by sight once their vision is restored? This question, posed by Molyneux in 1688, has puzzled philosophers and scientists since then. To overcome ethical and practical difficulties in the investigation of cross-modal recognition, we studied inexperienced poultry chicks, which can be reared in darkness until the moment of a visual test with no detrimental consequences. After hatching chicks in darkness, we exposed them to either tactile smooth or tactile bumpy stimuli for 24 h. Immediately after the tactile exposure, chicks were tested in a visual recognition task, during their first experience with light. At first sight, chicks that had been exposed in the tactile modality to smooth stimuli approached the visual smooth stimulus significantly more than those exposed to the tactile bumpy stimuli. These results show that visually inexperienced chicks can solve Molyneux's problem, indicating cross-modal recognition does not require previous multimodal experience. At least in this precocial species, supra-modal brain areas appear functional already at birth. This discovery paves the way for the investigation of predisposed cross-modal cognition that does not depend on visual experience.

## 1. Introduction

On 7 July 1688, William Molyneux posed a question that has puzzled philosophers and scientists ever since [1]: If a congenitally blind person learns to distinguish between a cube and a sphere by touch, would they immediately recognize these objects by sight, if their vision is restored? In other words, we ask whether it is possible to recognize objects by matching representations between two different sensory modalities, without the need for previous experience of both sensory modalities. To address this question, Molyneux's problem, and understand whether cross-modal recognition exists in the absence of cross-modal experience, methodological and ethical issues need to be addressed.

First, the role of previous experience must be excluded through deprivation of visual experience until the moment of test. While it is not possible, nor ethically desirable, to keep humans visually deprived from birth for experimental reasons, animal models can be a suitable experimental system. In particular, chicks are precocial, meaning that they hatch with mature visual, proprioceptive and motor systems, so that their perceptive and motor responses can be evaluated already in the first hours of life [2,3], avoiding long sensory deprivations that can impair normal development. Moreover, during this short time chicks do not require tactile interactions to survive, reducing both ethical issues and methodological confounds.

Another problem encountered in addressing Molyneux's problem that can be solved by studying precocial species is maintaining the functionality of sensory systems despite visual deprivation. When sight is restored after long-term blindness or in congenitally blind human patients, for instance via surgical removal of a cataract, sight can be impaired. For this reason, the lack of transfer from tactile discrimination to vision observed immediately after the onset of sight (from Cheselden 1728, to more recent examples [4]) can be due to misperception or degradation of the visual system. On the other hand, successful transfers [5] can be due to learning from verbal descriptions or associations from other sensory modalities (or to early experience [6]).

An alternative approach has focused on early life responses. Tactual–visual recognition has been found in human newborns exposed to pacifiers or objects with different shapes [7,8]. Yet, this early performance could not rule out the effect of previous experience. In contrast, the use of poultry chicks tested a few hours after hatching addresses these issues because the development of the somatosensory system is largely complete before hatching [9]. Second, chicks' vision is not impaired by temporary deprivation of visual experience, enabling experiments where subjects have no visual experience before their first visual test. Furthermore, investigating cross-modal recognition in non-human animals rules out any influence of verbal reports.

Due to the advantages of using precocial birds as a model, we addressed Molyneux's problem focusing on domestic chicks (*Gallus gallus*). In Experiment 1 we tested visual recognition of objects previously experienced in tactile modality. The experiment comprised three phases: hatching in darkness, tactile exposure in darkness and visual recognition test in an illuminated arena where stimuli could not be touched. We reasoned that, during the exposure phase, chicks could use tactile experience to learn the smooth versus bumpy tactile quality of the stimuli, similarly to what happens in visual filial imprinting. Filial imprinting [10] is a learning mechanism based on exposure, where young animals become attached to the objects they are exposed to, without any reinforcement. As a result, after imprinting exposure, chicks tend to approach familiar objects. This phenomenon has been widely documented in visual and acoustic modality [11,12] also for still objects [13]. Hence, differently from the original formulation of the problem, chicks experienced only one type of stimulus (the familiar one) during the exposure phase.

We hypothesized that if chicks could cross-modally learn the visual properties of objects via tactile experience, their subsequent visual choices would reflect tactile experience. In line with this hypothesis, the visual choices observed in Experiment 1 depended on previous tactile experience, with chicks tactily exposed to smooth stimuli choosing smooth stimuli significantly more than chicks tactily exposed to bumpy stimuli. To assess how chicks explore surrounding objects when hatched in darkness, we used infrared camera recordings (Experiment 2). We observed that chicks hatched in darkness spent a large portion of their time in contact with the tactile stimuli provided, a behaviour that enables tactile learning.

## 2. Methods

### (a) Experiment 1: cross-modal recognition

#### (i) Subjects and rearing conditions

**Figure 1.**
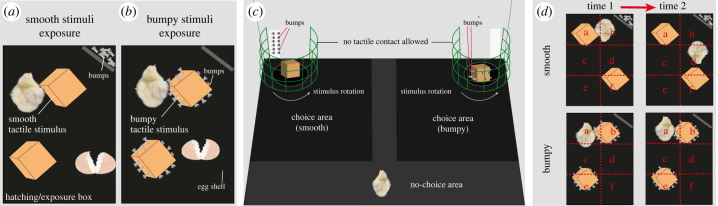
Experimental setting. In Experiment 1, hatching and tactile exposure compartments contained the smooth (*a*) and bumpy (*b*) stimuli that chicks experienced in darkness for 24 h. After tactile exposure, chicks were moved to the testing arena (*c*), where they could visually explore and approach the smooth and bumpy stimuli. The stimuli were located behind a mesh and could not be touched. For visual and olfactory matching, bumps (metal bolts) were present on both sides of the arena. In Experiment 2, the position of chicks in the tactile exposure compartments (*d*) was analysed considering the position in the a–f areas illustrated in the figure. For instance, in the top panel, the chick is in contact with the stimulus at Time 1 but not at Time 2, and is moving between Time 1 and Time 2. In the bottom panel, the chick is in contact with the stimulus at Time 1 and Time 2, and is not moving between Time 1 and Time 2.

Overall, we tested 103 chicks (55 females, 48 males) (*Gallus gallus*) of the Ross 308 strain, and 72 chicks (39 females, 33 males) made a choice, entering one of the choice areas of the apparatus (figure 1*c*), during the test and were analysed. Among the chicks that did not make a choice, 12 (7 females, 5 males) had been exposed to the bumpy stimuli and 19 (9 females, 10 males) to the smooth stimuli.

Fresh eggs were collected from Avara Foods (Rayne, UK), incubated in darkness at 37.7° C and 40–60% humidity in a FIEM MG-140 incubator for 18 days. At day 18 of incubation, eggs were moved to individual hatching compartments (28 × 18 × 11 cm) in a FIEM MG-316 hatchery. Incubation and hatching happened in complete darkness.

#### (ii) Stimuli

Tactile stimuli were wooden cubes (5 cm), with or without metal bolts (16 mm stainless steel) drilled on the sides as bumps. Bumpy stimuli had three bolts on each side wall (12 bolts for each stimulus), protruding 10 mm (figure 1*a,b*). Since smooth stimuli had no bolts on the sides, 24 bolts were located in a separate subsection of each compartment. Previous research showed that metal does not smell [14], but this equalized the quantity of material between conditions. The bolt subsection was left empty in the bumpy condition.

#### (iii) Apparatus

For the test, we used a rectangular wood arena (90 × 60 × 52 cm) with white walls and a black non-slip mat floor (figure 1*c*). The two different tactile stimuli were placed on two corners of the arena, on black rotators (15.1 cm diameter) that moved at 2.4 rotations/minute on the same clockwise/anticlockwise direction. The right–left position of the stimuli was counterbalanced between chicks. To prevent tactile interactions with the stimuli, a green plastic mesh surrounded each rotator. To present the same visual material in both corners, we located an empty plastic box either behind the bumpy stimulus and a plastic box with 12 visible bolts drilled behind the smooth stimulus.

A LED strip above the apparatus illuminated the arena. We recorded the experimental sessions with a Microsoft LifeCam Studio webcam (10 fps) located above the centre of the arena. For analysing chicks' preferences, the arena was virtually divided in three areas: a left and a right area close to the stimuli (40 × 40 cm) and a no-choice area (figure 1*c*).

#### (iv) Experimental procedure

Chicks hatched in darkness in individual compartments that contained either two smooth or two bumpy stimuli (figure 1*a,b*). Chicks remained for 24 h in their hatching box, where they could explore the smooth or bumpy stimuli through tactile sensory modality. The exposure phase lasted 24–30 h, after which chicks were individually tested. Each chick was moved into an individual opaque box, covered with a lid, and gently transported to the testing room. At test, each chick was sexed and gently located in the illuminated testing arena, in the no-choice area opposite to the stimuli (figure 1*c*). This was their first experience with visual stimuli. Chicks were observed and their position analysed for 6 min. Chicks that did not enter any side areas within 6 min were not included in statistical analyses, in line with previous literature [12,13,15,16].

After tactile exposure in darkness, chicks were individually transported to the testing room in an opaque box and presented with a visual recognition task of the smooth versus bumpy stimuli (electronic supplementary material, movie S1). Chicks could freely move in the arena.

#### (v) Video and data analysis

We assessed the preferences of chicks for the smooth versus bumpy object using the position of their centroid, based on the number of frames spent in each area. We tracked the chicks' position using the open-source system DeepLabCut [17]. For the centroid of each chick, we ensured that the quality of tracking reached an accuracy of 0.9 likelihood for at least 90% of frames, since this threshold ensured high accuracy. For each of the six 1 min time bins, the Preference for the smooth stimulus was calculated as: (Time spent in the smooth stimulus area)/(Total time spent in the smooth + bumpy stimulus areas) × 100. The preference for the bumpy stimulus is the reciprocal of the preference for the smooth stimulus. For instance, if the preference for the smooth stimulus is 60%, the preference for the bumpy stimulus is 100 − 60 = 40%.

To compare the choices of chicks previously exposed to smooth versus bumpy tactile stimuli we ran a mixed design ANOVA with Preference for smooth stimulus as dependent variable, Exposure condition (smooth or bumpy) and Sex (female or male) as between-subjects variables and Time bin (1–6) as within-subjects variable. Alpha was set to *p* < 0.05. We used R [18] (packages: ggplot2, rstatix, plyr) for statistical analysis and plots.

### (b) Experiment 2: tactile experience in darkness

#### (i) Subjects, incubation, hatching, stimuli

We tested 34 chicks (15 females, 19 males) (G*allus gallus*) of the Ross 308 strain, collected, maintained, incubated and hatched in the same conditions and with the same stimuli described in the previous experiment. Because the hatcheries were too small to host infrared cameras, recordings of the activity of chicks during exposure to the tactile stimuli were conducted on different chicks from those tested in the previous experiment, in a pre-warmed (30–32°C) room, in full darkness. A double door system prevented any light filtering in the room. The same hatching/exposure compartments described in the previous experiment were used. We recorded the chicks’ movement using a Foscam C2M infrared camera.

#### (ii) Experimental procedure, video and data analysis

After hatching in the tray with tactile stimuli, chicks were individually moved from the hatchery to an identical compartment located in a pre-warmed room. For the transport, chicks were located in individual opaque boxes in darkness, and boxes were located in an opaque bag. Chicks were then gently located in a hatching/exposure tray and recorded for 24 h.

For the analysis of chicks' position, we virtually divided the rectangular area of the exposure compartment into six squares (a–f, figure 1*d*) and used scan sampling to measure the percentage of time in which a chick was moving (centroid located in two different areas between two subsequent time points) and the percentage of time in which chicks were in touch with a tactile stimulus. We assessed chicks’ behaviour for 5 min every hour (2 out of 24 h of recording), sampling at 20 s intervals (15 sampling times) in each hour. We estimated the average percentage of time that each chick spent moving as: (Number of observations in which the chick's centroid changed position)/(Total number of observations) × 100. We estimated the average percentage of time that each chick spent moving as: (Number of observations in which the chick's centroid changed position)/(Total number of observations) × 100.

## 3. Results

**Figure 2.**
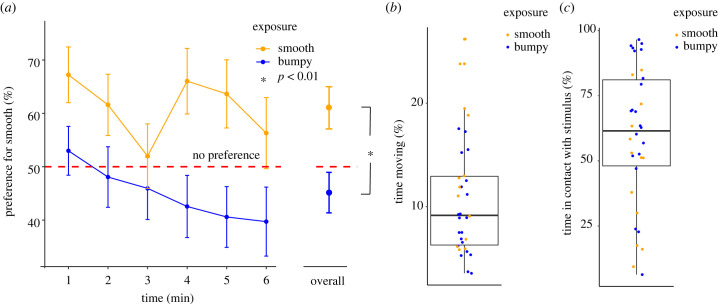
Panel (*a*) shows the preference for the smooth stimulus (mean ± SEM) during the 6 min of the test, in chicks previously exposed to the smooth (orange) and bumpy (blue) stimuli. The overall preference is shown on the right side, with chicks exposed to smooth tactile stimuli choosing visual smooth stimuli, and chicks exposed to bumpy tactile stimuli choosing bumpy visual stimuli. In Experiment 2, newly hatched chicks spent about 10% of the time moving (*b*) and about 50% of their time in touch with the tactile stimuli (*c*).

In Experiment 1, during their first 6 min of visual experience chicks exposed to the smooth stimuli spent more time close to the smooth stimuli compared to chicks exposed to the bumpy stimuli (*F*_1,68_ = 9.026, *p* = 0.004, *M*_smooth_ = 61.13, SEM_smooth_ = 4.00; *M*_bumpy_ = 44.98, SEM_bumpy_ = 3.98; figure 2*a*). This solid effect (in line with the magnitude of imprinting responses for visual stimuli [12,13]) shows that tactile exposure is effective to influence visual recognition tests. No significant differences between sexes and time points or interactions were detected. Already in the first minute of the test, the group exposed to the smooth stimuli had a visual preference for the smooth stimulus significantly higher than the group exposed to tactile bumpy stimuli (*t*_67.60_ = −2.05, *p* = 0.044).

In Experiment 2, infrared camera recordings showed that chicks hatched in darkness explored their surroundings, moving about 10% of their time (*M* = 10.78%, s.d. = 5.65, median 9.17%; Smooth object condition: *M* = 13.15%, s.d. = 6.91, median = 11.90%; Bumpy object condition: *M* = 9.33%, s.d. = 4.26, median = 11.90; figure 2*b*). Moreover, we observed that chicks spent the majority of their time (about 60%, > 14 h) in contact with the tactile stimuli: *M* = 59.07%; s.d. = 26.45, median 61.51%; Smooth object condition: *M* = 48.28% (about 12 h), s.d. = 24.84, median = 51.21%; Bumpy object condition: *M* = 65.75% (greater than 15 h), s.d. = 25.73, median = 68.89, figure 2*c*. These results show that in all conditions chicks had plenty of direct tactile exposure to imprint in tactile modality.

## 4. Discussion

To understand whether tactile to visual cross-modal recognition is possible without previous cross-modal experience (Molyneux's problem), we tested whether the visual choices of visually naive chicks, previously exposed in tactile modality to either smooth or bumpy cubes, are driven by tactile experience. At their first visual experience, chicks previously exposed to tactile smooth stimuli approached the visually smooth stimulus significantly more than chicks previously exposed to the tactile bumpy stimuli. The difference appeared at first sight, already in the first minute of the test. This significant difference in visual preferences in chicks that differed only in tactile experience shows that visually naive chicks learn about objects experienced in the solely tactile modality, and can use representations based on tactile experience to solve a visual recognition task. The ability of newly-hatched chicks to discriminate between visual objects at first sight, based on previous tactile experience, solves Molyneux's problem, showing that cross-modal recognition from tactile to visual sensory modality does not require previous experience with simultaneous multi-modal stimuli.

Infrared camera recordings showed that, in darkness, newly hatched chicks moved and spent most of their time in touch with the tactile stimuli. This is not surprising because tactile experience in darkness is a common experience for newly hatched chicks in the wild, given that they spend the first 3 days of life mainly under the mother hen [19]. These results suggest that chicks might be able to imprint in tactile sensory modality. Further research should clarify whether chicks exhibit tactile predispositions that facilitate approach and imprinting responses [2,3,20].

The empiricist John Locke and his followers thought that Molyneux's question could not be solved without previous experience [1]. Indeed, previous attempts to address the question focused on adult animals that had previously experienced simultaneous experience with the two sensory modalities under test (touch and vision in bumblebees [21], vision and active electric sense in a weakly electric fish [22]). This design did not exclude that animals had learnt to match features between sensory modalities through previous simultaneous multimodal experience. Here, we have shown spontaneous cross-modal recognition in the absence of previous cross-modal and multimodal experience. We focused on visual to tactile cross-modal recognition only, because chicks show limited exploration of the environment in darkness.

By answering a centuries-old philosophical problem, newly-hatched chicks contradict this claim, showing that the brain is equipped to spontaneously match visual and tactile information at birth. This evidence suggests that cross-modal or supra-modal brain areas [23,24] might already be functional at birth, at least in this precocial species, without the need of experience and multi-modal associations. This discovery paves the way to investigation of predisposed cross-modal cognition that does not depend on visual experience.

## Data Availability

All data are available in the main text or the electronic supplementary material [25].
